# Roles of Long Noncoding RNAs in Conferring Glioma Progression and Treatment

**DOI:** 10.3389/fonc.2021.688027

**Published:** 2021-06-11

**Authors:** Jie Qin, Chuanlu Jiang, Jinquan Cai, Xiangqi Meng

**Affiliations:** Department of Neurosurgery, The Second Affiliated Hospital of Harbin Medical University, Harbin, China

**Keywords:** lncRNAs, glioma, biomarker, therapeutic target, prognosis, chemoresistance

## Abstract

Accompanying the development of biomedicine, our knowledge of glioma, one of the most common primary intracranial carcinomas, is becoming more comprehensive. Unfortunately, patients with glioblastoma (GBM) still have a dismal prognosis and a high relapse rate, even with standard combination therapy, namely, surgical resection, postoperative radiotherapy and chemotherapy. The absence of validated biomarkers is responsible for the majority of these poor outcomes, and reliable therapeutic targets are indispensable for improving the prognosis of patients suffering from gliomas. Identification of both precise diagnostic and accurate prognostic markers and promising therapeutic targets has therefore attracted considerable attention from researchers. Encouragingly, accumulating evidence has demonstrated that long noncoding RNAs (lncRNAs) play important roles in the pathogenesis and oncogenesis of various categories of human tumors, including gliomas. Nevertheless, the underlying mechanisms by which lncRNAs regulate diverse biological behaviors of glioma cells, such as proliferation, invasion and migration, remain poorly understood. Consequently, this review builds on previous studies to further summarize the progress in the field of lncRNA regulation of gliomas over recent years and addresses the potential of lncRNAs as diagnostic and prognostic markers and therapeutic targets.

## Introduction

Gliomas, originating from glial or precursor cells, which are categorized into astrocytomas, ependymomas and oligodendrogliomas, are the most common malignant primary tumors of the central nervous system (CNS) (1, 2). In addition, gliomas are graded by the World Health Organization (WHO) into four classifications based on their malignancy. Gliomas with WHO grades I-II are known as low-grade gliomas (LGGs), including angiocentric glioma and diffuse astrocytoma, while those with WHO grades III-IV are considered high-grade gliomas (HGGs), including mesenchymal astrocytoma and glioblastoma multiform gliomas (GBMs) (3, 4). In the 2016 WHO classification of CNS tumors, molecular parameters, including IDH, ATRX, TP53 and 1p/19, were considered in the classification of glioma subtypes, which is more detailed than its 2007 predecessor (5, 6). GBM has high mortality and recurrence rates and represents the most malignant CNS tumor (3). The present criteria for treating GBM continue to be neurosurgical resection of the neoplasm accompanied by chemotherapy with temozolomide (TMZ) and radiotherapy (7). Unfortunately, the median survival for GBM patients is only 15 months, even with this combination treatment (8). Therefore, exploring the specific mechanisms of the occurrence and progression of glioma has drawn widespread interest in recent years. Studies on precise biomarkers and reliable therapeutic targets are urgently needed.

Approximately 98% of transcripts do not encode proteins in the human genome, and this category of RNA is known as noncoding RNA (ncRNA). Long noncoding RNAs (lncRNAs), accounting for approximately 80-90% of ncRNAs, are transcripts consisting of more than 200 nucleotides that typically lack protein-coding capability and were once regarded as transcriptional noise (9). Open reading frames are generally absent in lncRNAs (10). Intriguingly, this “transcriptional noise” has been extensively researched and demonstrated to not only serve an important function in normal cellular physiological procedures but also play an invaluable role in regulating the malignant behavior of tumors (11). LncRNAs can be divided into sense lncRNAs, antisense lncRNAs, bidirectional lncRNAs, intronic lncRNAs and intergenic lncRNAs (LINCRNAs) based on genomic location (12). The order of nucleotide arrangement constitutes the primary structure of lncRNAs, and intricate secondary and tertiary structures guarantee the multiple functions of lncRNAs. However, the relationship between lncRNA secondary structure and functions remains unclear. Recent evidence has indicated that lncRNAs regulate gene expression at three levels: transcriptional, post-transcriptional and epigenetic modification (13).

As mentioned above, lncRNAs are associated with both cellular physiology and disease origination and progression by regulating gene expression (11). Additionally, an increasing number of investigations have suggested that lncRNAs play pivotal roles in regulating the tumorigenesis, proliferation, aggression, metastasis, and drug resistance of gliomas. Consequently, as the molecular mechanism of lncRNA regulation of glioma is further investigated, the etiology of glioma will gradually be revealed. Furthermore, along with the advancement of sequencing technology, we will gradually recognize the entire spectrum of lncRNAs, implying that lncRNAs could be not only effective indicators for early diagnosis and determination of prognosis but also therapeutic targets for glioma.

## The Functions and Mechanisms of lncRNAs in Gliomas

**Figure 1:**
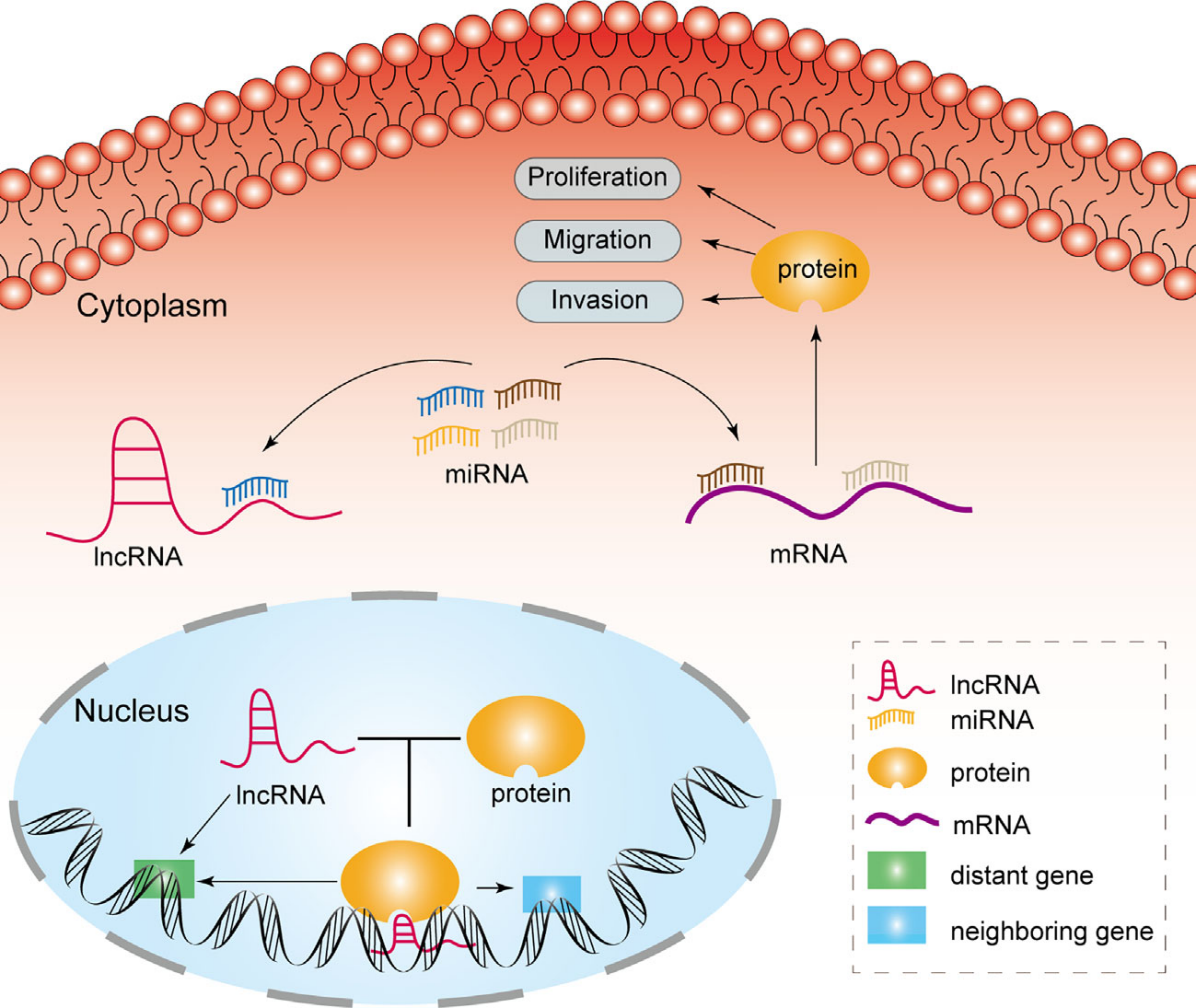
Mechanisms of lncRNAs in glioma cell.

MiRNAs are a category of noncoding RNAs of approximately 20 nucleotides in length that can bind to target mRNAs *via* microRNA response elements (MREs) and thus perform negative regulatory functions, exerting critical post-transcriptional regulatory effects (14). MREs are short sequences of both lncRNAs and mRNAs that combine with miRNAs. Therefore, lncRNAs absorb miRNAs as sponges, enabling the expression of mRNAs that were previously repressed by miRNAs, and such lncRNAs are referred to as competitive endogenous RNAs (**Figure 1**) (15). Many studies have been conducted to demonstrate that lncRNAs, as ceRNAs, impact the progression of tumors at the post-transcriptional regulatory level (**Table 1**).

**Table 1:** The role of lncRNAs as ceRNA in the glioma.

LncRNA	MiRNA	Expression of mRNA	Function	Study
LINC00689	miR-338-3p	Upregulated PKM2	Promoting growth, metastasis and glycolysis	16
LINC01857	miR-1281	Upregulated TRIM65	Promoting growth, migration, and invasion	17
MALAT1	miR-199a	Upregulated ZHX1	Promoting proliferation and progression.	18
SNHG1	miR-194	Upregulated PHLDA1	Promoting progression	19
BLACAT1	miR-605-3p	Upregulated VASP	Promoting progression	20
AC016405.3	miR-19a-5p	Upregulated TET2	Acting as tumor suppressor	21
GACAT3	miR-135a.	Upregulated NAMPT	Promoting progression	22
MATN1-AS1	miR-200b/c/429	Upregulated CHD1	Promoting progression	23
TPT1-AS1	miRNA-770-5p	Upregulated STMN1	Inhibiting autophagy and promoting proliferation	24
LEF1-AS1	miR-489-3p	Upregulated HIGD1A	Promoting tumorigenesis	26
NCK1-AS1	miR-138-2-3p	Upregulated TRIM24	Promoting tumorigenesis	28
AGAP2-AS1	miR-15a/b-5p	Upregulated HDGF	Promoting proliferation	29
SNHG16	miR-373	Upregulated EGFR	Promoting tumorigenicity	30
LINC00475	miR-449b-5p	Upregulated AGAP2	Acting as a tumor suppressor	41
HCG11	miR-496	Upregulated CPEB3	Promoting progression	43
NEAT1	miR-139-5p	Upregulated CDK6	Promoting proliferation, invasion and migration	45
NEAT1	miR-107	Upregulated CDK14	Promoting progression	52
BCYRN1	miR-619-5p	Upregulated CUEDC2	Inhibiting tumorigenesis	50
LINC00174	miR-152-3p	Upregulated SLC2A1	Promoting glycolysis and tumor progression	51
LPP-AS2	miR-7-5p	Upregulated EGFR	Promoting tumorigenesis	54
LINC00645	miR-205-3p	Upregulated ZEB1	Promoting epithelial-mesenchymal transition (EMT)	55
HOTAIR	miR-148b-3p	Upregulated USF1	Regulating blood-tumor barrier (BTB) permeability	58
MIAT	miR-140-3p	Upregulated ZAK	Regulating BTB permeability	59
Lnc00462717	miR-186-5p	Upregulated PTBP1	Regulating BTB permeability	61
LINC00174	miR-138-5p/miR-150-5p	Upregulated FOSL2	Regulating BTB permeability	62
TALC	miR-20b-3p	Upregulated c-Met	Promoting MGMT expression	66
SNHG15	miR-726	Upregulated CDK6	Overcoming temozolomide (TMZ) resistance	68
AC003092.1	miR-195	Upregulated TFPI-2	Promoting TMZ chemosensitivity	71
CASC2	miR-181a	Upregulated PTEN	Promoting glioma growth and resistance to TMZ	72
SNHG16	miR-212-3p	Upregulated USF1	Promoting vasculogenic mimicry	78
LINC00667	miR-429	Upregulated USF1	Promoting vasculogenic mimicry	78
SNHG1	miR-154-5p/miR-376b-3p	Upregulated FOXP2	Promoting growth, migration, and invasion	39
PDIA3P1	miR-124-3p	Upregulated RELA	Promoting EMT	83
LINC01579	miR-139-5p	Upregulated EIF4G2	Promoting proliferation	84

By analyzing GSE4290, Liu and colleagues showed that LINC00689 was highly expressed in glioma tissue compared to normal brain tissue. The expression of pyruvate kinase M2 (PKM2) was enhanced by LINC00689-mediated elimination of miR-338-3p, which facilitated malignant progression of glioma cells. As a consequence, the LINC00689/miR-338-3p/PKM2 axis functions as a carcinogenic driver in gliomas (16). Moreover, LINC01857 could promote tumorigenesis of glioma by sponging miR‐1281 to upregulate TRIM65 expression (17). MALAT1 has been proved to have a crucial role in the progression of multiple neoplasms such as lung, colorectal and gastric cancers, and shows a comparable regulatory role in glioma. MALAT1 promoted the level of ZHX1 by serving as a ceRNA of miR-199a, leading to augmented glioma development (18). LINC01579 accelerated cell proliferation and apoptosis of GBM by the competitive binding of miR-139-5p to affect EIF4G2 (19). Furthermore, the lncRNA SNHG1 is considered a sponge that absorbs miR-194 to promote glioma progression by regulating PHLDA1 expression (20). MiR-605-3p was eliminated by lncRNA BLACAT1 to accelerate VASP expression, contributing to glioma proliferation (21). The expression level of NAMPT was regulated by lncRNA-GACAT3 to promote glioma progression as a sponge for miR135a (22). LncRNA MATN1-AS1 competitively binding with miR-200b/c/429 also promoted the progression of glioma by modulating CHD1 expression (23). Chai et al. demonstrated that exosomal lncRNAe-ROR1-AS1 enhanced glioma progression by suppressing miR-4686 (24). LEF1-AS1 promoted glioma formation by competitively binding miR-489-3p to increase the expression of HIGD1A (25). Oncogenic lncRNA FOXD1-AS1 promoted the proliferation and metastasis of GBM cells by targeting miR339/342 (26). However, lncRNA could also act as a repressor to inhibit tumor progression. Zhen et al. indicated that NEAT1 sponged miR-107 to inhibit the expression of cyclin-dependent kinase 14 (CDK14) to repress the malignant progression of glioma (27). The anti-oncogene AC016405.3 restrained GBM cell proliferation and migration by sponging miR-19a through regulation of ten-eleven translocation-2 (TET2) (28). LncRNA TPT1-AS1 inhibited glioma cell autophagy by decreasing the expression of miR-110-5p, and upregulating STMN1 expression promoted the proliferation of glioma cells (29). In addition, mRNAs can indirectly regulate gene phenotypes by signaling pathways *via* the post-transcriptional regulation of ceRNAs. For instance, miR-183-2-3p was sponged by lncRNA NCK1-AS1. Low levels of miR-183-2-3p promoted TRIM24 expression and thereby activated the Wnt/β-catenin pathway to contribute to glioma progression (30). Correspondingly, the lncRNA AGAP2-AS1 exhibited analogous mechanisms in contributing to the development of glioma advancement *via* the miR-15a/b-5p/HDGF/WNT axis (31). The oncogene lncRNA SNHG16, in contrast, functioned in the proliferation, aggression and migration of glioma cells through the miR373/EGFR/PI3K/AKT axis (32). These researches illustrated that lncRNAs could not only promote but also inhibit tumor progression.

By interacting with signaling pathways, lncRNAs can facilitate tumorigenesis. For example, silencing lncRNA MIR22HG inhibited GBM aggressiveness by suppressing the Wnt/β-catenin signaling pathway (33), while cancer susceptibility candidate 7 (CASC7) restrained the progression of glioma through the Wnt/β-catenin pathway (34). BCAR4 promoted glioma cell progression by stimulating the EGFR/PI3K/AKT pathway (35). LncRNA LPP-AS2 plays an important role in regulating the miR-7-5p/EGFR/PI3K/AKT/c-MYC feedback loop, which is correlated with glioma tumorigenesis (36). LncRNA BCYRN1 could suppress tumorigenesis of glioma as a molecular sponge of miR-619-5p to modulate the PTEN/AKT/p21 pathway and CUEDC2 expression (37). Furthermore, lncRNA MT1JP suppressed proliferation, invasion, and migration and promoted apoptosis of glioma cells through stimulation of the PTEN/Akt signaling pathway (38). LncRNA-THOR silencing accelerated human glioma cell apoptosis by activating the MAGEA6-AMPK signaling pathway (39). Accumulating studies have suggested that lncRNA is involved in the regulation of diverse biological behaviors in glioma through the regulation of signaling pathways including but not limited to Wnt/β, PI3K/AKT and NF-κB. Therefore, lncRNAs are promising biomarkers for glioma diagnosis, prognosis and treatment in theory.

**Figure 2:**
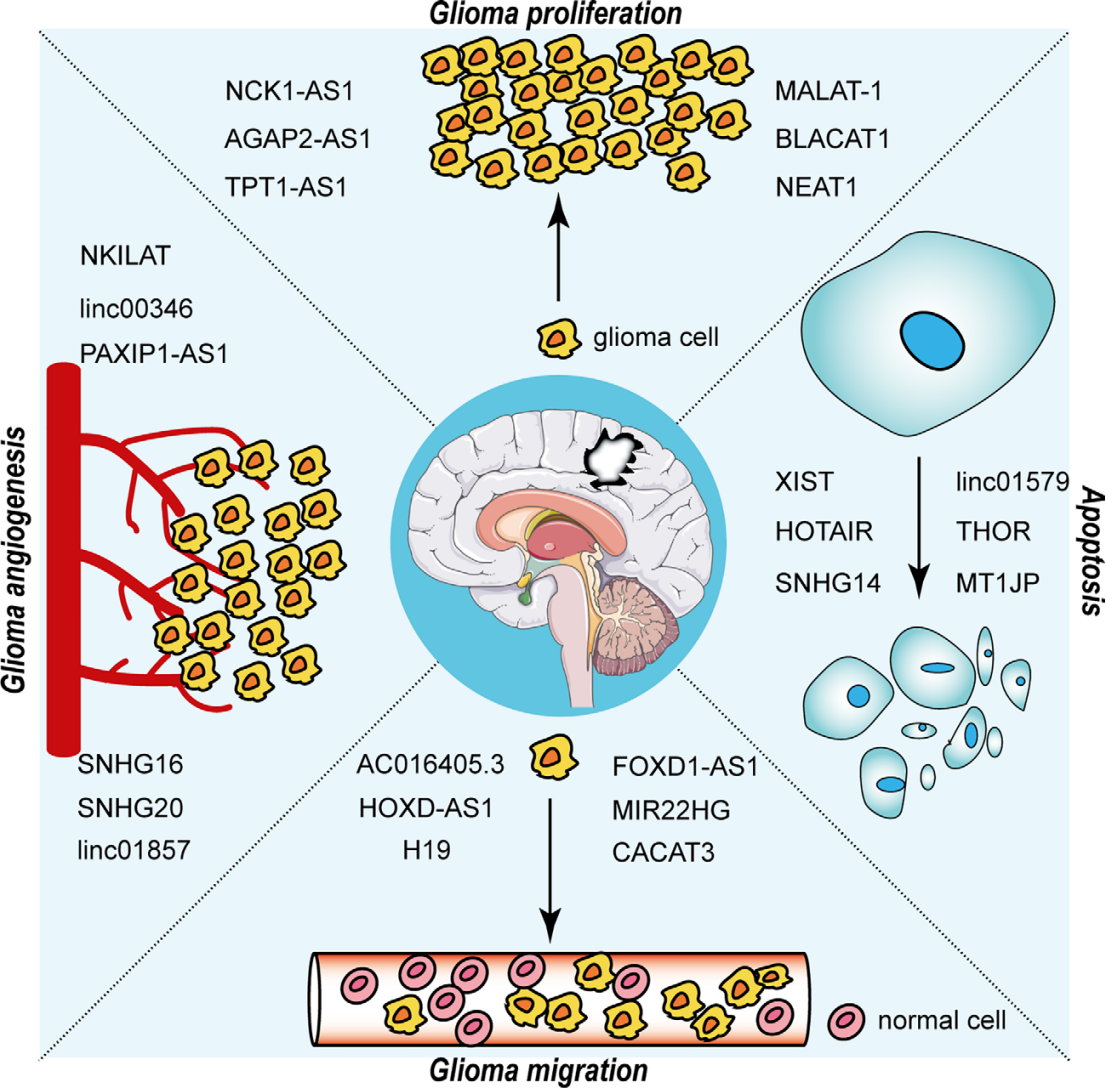
LncRNAs in the proliferation, migration, apoptosis and angiogenesis of glioma.

The interaction between RNA-binding proteins (RBPs) and lncRNAs plays a non-negligible role in the advancement of glioma. The expression level of EZH2 positively correlated with the malignancy of glioma and promoted the malignant behavior of glioma (40). Chen et al. first addressed the mechanism of the participation of lncRNA NEAT1 in tumorigenesis as a scaffold for EZH2. LncNEAT1 recruited EZH2 to interact with the promoter regions of downstream genes (Axin2, ICAT, GSK3B) to promote trimethylation modification of H3K27, thereby silencing these three genes. Further, the WNT/β-catenin pathway was activated, resulting in tumorigenesis (41). Moreover, RBP DGCR8 could bind with ZFAT-AS1, the interaction between DGCR8/ZFAT-AS1 and CDX2 contributed to the malignant progression of glioma (42). RBP, lncRNA and downstream gene could form negative or positive feedback loop to modulate biological behavior of glioma. SNHG1 regulated the miRNA154-5p/miR-376b-3p-FOXP2-KDM5B positive feedback loop to promote the malignant phenotype of glioma cells (43). LINC00475 silencing acted as a tumor suppressor in glioma under hypoxic conditions by impairing miRNA-449b-5p-dependent upregulation of AGAP2 expression (44). TRPM2‐AS inhibited the growth, migration, and invasion of gliomas through JNK, c‐Jun, and RGS4 (45). HCG11 inhibited glioma progression by modulating miR‐496 to upregulate cytoplasmic polyadenylation element binding protein 3 (CPEB3) expression (46). The lncRNA MNX1-AS1 reduced the level of miR-4443, leading to the promotion of proliferation, invasion and migration in glioma (47). NEAT1 and CDK6 could promote tumorigenesis of glioma cells; additionally, miR‐139‐5p restrained the biological functions of glioma cells (48). LncRNAs has diverse roles in glioma processes, such as proliferation, migration, apoptosis and angiogenesis, by distinct mechanisms, including ceRNA, interaction with RBPs and regulation of mRNA. **Figure 2** portrays the lncRNAs associated with glioma proliferation, metastasis, apoptosis and angiogenesis.

## LncRNAs as Diagnostic and Prognostic Biomarkers of Gliomas

Medical diagnosis in the twenty-first century is gradually moving from clinical pathology to molecular pathology. With the development of bioinformatics, an increasing number of studies have identified lncRNAs as biomarkers for glioma diagnosis and prognosis by exploring RNA microarrays, and advances in microarray and high-throughput RNA-seq technologies have provided numerous valuable lncRNAs for the diagnosis and prognosis of gliomas.

The detection of serum HOTAIR levels can be employed for the clinical diagnosis of glioma, as reported by Tan et al. (49) These researchers also observed that the serum levels of HOTAIR were significantly higher in GBM patients than in controls, with a sensitivity of 86.1% and specificity of 87.5% (49). This report first showed that HOTAIR can function as a novel diagnostic and prognostic peripheral biomarker of GBM. Lin et al. screened six lncRNAs associated with low-grade glioma prognosis by TCGA and GTEx RNA-seq databases. These researchers constructed a prognostic risk signature with 6 lncRNAs in LGG, and this research illustrated that AL031722.1 and LINC00844 decreased when the risk score was increased, while the expression of AL354740.1, FGD5-AS1, and NEAT1 increased (50). The team of Li et al. indicated that the expression of LINC01060 was upregulated in glioma and significantly related to tumor grade and poor clinical prognosis (51). Furthermore, Liu et al. revealed that the level of RMST was related to histological grade, and 95.6% of HGGs had higher RMST expression (52). The lncRNA HOTAIRM1 was identified as a prognostic factor for glioma because it can maintain the tumorigenicity of GSCs by regulating HOX gene expression (53). And, LINC00115 was shown to act as a key role in GSC self-renewal and tumorigenicity by Tang et al. (54) LINC00174 accelerated glycolysis and tumor progression by competitively binding with miR-152-3p in glioma, indicating this molecule might act as a molecular target for glioma diagnosis (55). LncRNA H19, which mediates the effect of curcumin in treating glioma accompanied by miR-675 and VDR, could act as a novel diagnostic biomarker (56). Li et al. showed that LINC00645 could promote EMT, which was indispensable in the invasion and migration of glioma cells involving TGF-β by regulating the miR-205-3p-ZEB1 axis; thus, LINC00645 could be a prognostic indicator for glioma (57). A novel lncRNA-TOCN that targets the Smad2/PKCα signaling pathway to inhibit malignant progression of glioma was highlighted by Tang et al. and could serve as a prognostic indicator of GBM (58). The expression of multiple lncRNAs has been documented to correlate with the malignancy of gliomas and to be involved in their malignant progression, providing powerful theoretical evidence for their application as diagnostic and prognostic markers.

## LncRNAs as Reliable Therapeutic Targets

### Treatment Strategies Involving LncRNAs as Regulators Modulating the BTB

The BTB parallels the blood-brain barrier (BBB) and is comprised of vascular endothelial cells, basement membrane, and glioma cells. This structure can seriously impede the entry of drugs into the tumor microenvironment, resulting in poor drug efficacy and extremely unfavorable patient prognosis (59–61). Consequently, exploring lncRNAs that can regulate BTB permeability to promote chemotherapy and thus improve drug efficacy is one of the research directions for targeted glioma therapy. Li et al. showed that silencing HOTAIR could increase BTB permeability by eliminating miR-148b-3p, thereby further reducing the expression of glioma-microvascular endothelial cell tight junction (TJ)-related proteins by targeting USF1 (62). He et al. also indicated that MIAT regulated the expression of ZAK to promote the delivery efficiency of doxorubicin across the BTB (63). In addition, the IGF2BP2/FBXL19-AS1/ZNF765 axis could regulate the permeability of the BTB to improve the antitumor effect of doxorubicin (64). Lnc00462717 regulated BTB permeability by interacting with PTBP1 to restrain the miR-1865p/Occludin signaling pathway (65). Moreover, BTB permeability was shown to be augmented by silencing LINC00174 in glioma tissue (66). Overcoming the obstacle of the BTB to increase the local concentration of chemotherapeutic agents in glioma and then enhance therapeutic efficacy is a prospective strategy. As such, identifying appropriate targets has been a major concern. The formulation of individual drug delivery routes based on the corresponding targets is another strategy that can potentially enhance chemotherapeutic efficacy.

### Treatment Strategies Involving LncRNAs Overcoming TMZ Resistance

Temozolomide (TMZ) is an oral alkylating agent that passes through the BBB, adding methyl groups to the purines of DNA to cause DNA damage and apoptosis for therapeutic effects (67, 68). Conversely, this process is reversed by the DNA damage repair enzyme O^6^-methyl-guanine-DNA methyltransferase (MGMT), which restores the damage caused by TMZ, resulting in the resistance of glioma cells to TMZ (69). A novel lncRNA, lnc-TALC, was found to be highly expressed in TMZ-resistant GBM cells by Wu et al. Lnc-TALC modulated the c-Met pathway by functioning as a ceRNA for miR-20b-3p, facilitating MGMT performance and in turn leading to TMZ resistance in GBM cells (70). Sun and colleagues revealed that the overexpression of miR-29c-3p could promote chemosensitivity to cisplatin, and CRNDE, which competitively binds with miR-29c-3p, plays a critical role in regulating the chemoresistance of medulloblastoma (71). In addition, tumorigenesis of glioma was attenuated with the downregulation of lncRNA-SNHG15 expression, and TMZ sensitivity was increased (72). Recently, a study reported that combining p50 and p53 with the proximal κB and p53 sites of the MALAT1 coding region, respectively, cooperatively downregulated MALAT1 expression, which in turn increased the chemosensitivity of GBM cells (73). The tumor microenvironment was remodeled with the secretion of oncogenic lncSBF2-AS1-enriched exosomes by GBM cells, resulting in tumor drug resistance (74). The lncRNA AC003092.1 inhibited miR-195, increasing the expression of tfpi-2, which promoted TMZ-induced apoptosis and thus made GBM cells more sensitive to TMZ (75). CASC2 has an essential function in the sensitivity of glioma to TMZ by upregulating PTEN expression through direct inhibition of miR-181a (76). High expression of SNHG12 in TMZ-resistant cells served as a molecular sponge for miR-129-5p to raise the levels of MAPK1 and E2F7 to enhance the sensitivity of GBM cells to TMZ. In contrast, knockdown of SNHG12 restored TMZ sensitivity (77). LncRNA SOX2OT activates the Wnt5a/β-catenin signaling pathway through upregulation of SOX2 expression, thereby inhibiting apoptosis, promoting cell proliferation, and resulting in resistance to TMZ (78). LncRNAs are not only linked to chemotherapy but also closely correlated with radioresistance. For instance, LINC-RA1 inhibited autophagy and enhanced radioresistance by inhibiting the H2Bub1/USP44 combination in glioma cells (79). In summary, lncRNA on the one hand can increase sensitivity of glioma to TMZ and on the other hand induce TMZ resistance of glioma. TMZ is currently the main chemotherapeutic agent for the treatment of glioma, however, glioma is prone to become resistance to it. Thus, it is feasible to target lncRNA to find drugs to overcome TMZ resistance in glioma.

### Treatment Strategy Involving LncRNAs Mediating Angiogenesis

Angiogenesis is a requirement for the growth and metastasis of gliomas, which are solid tumors. Additionally, extensive evidence has demonstrated that the formation of novel blood vessels participates in the development and metabolic processes of tumors. Therefore, vasculogenic mimicry is considered a hallmark of malignant tumor development. Hence, antiangiogenic treatment is anticipated to be an additional efficacious strategy for glioma. Chen and colleagues demonstrated that overexpression of NKILAT was negatively correlated with survival time in glioma patients, and NKILAT augmented the Warburg effect and angiogenesis in glioma, suggesting that it may be a promising therapeutic strategy (80). In addition, Yang et al. elucidated the key role of the ANKHD1/LINC00346/ZNF655 feedback loop in regulating angiogenesis in glioma (81). Likewise, Wang et al. demonstrated that knockdown of USF1 suppressed angiogenesis in gliomas by stressing SNHG16/miR-212-3p and the LINC00667/miR-429 axis (82). Furthermore, overexpression of lncRNA PAXIP1-AS1 promoted glioma vasculogenic mimicry by recruiting the transcription factor EST to upregulate KIF4 expression (83). SNHG20 played a crucial role in the ZRANB2/SNHG20/FOXK1 axis to regulate vasculogenic mimicry of glioma (84). These studies provide compelling evidence that lncRNAs potentially act as therapeutic targets by regulating angiogenesis in gliomas.

In addition, lncRNAs that exert regulatory effects by binding to RBP also have the potential to become therapeutic targets. Lin28a elevated the expression and stability of SNHG14, while deletion of SNHG14 increased the expression of IRF6, which inhibited the transcription of PKM2 and GLUT1 and thus impaired glycolysis and proliferation of glioma cells and induced apoptosis. Therefore, considering the lin28a/SNHG14/IRF6 axis as a target provides novel insight for the treatment of glioma (85). Additionally, the TAF15/LINC00665/MTF1(YY2)/GTSE1 axis is crucial for regulating the malignant biological behaviors of glioma cells, which might help in the development of a novel therapeutic strategy for human glioma (86). The PABPC1-BDNF-AS-RAX2-DLG5 axis was shown to play the same role as the TAF15/LINC00665/MTF1(YY2)/GTSE1 axis in regulating the biological behavior of gliomas (87). LncRNA PDIA3P1 promoted glioma mesenchymal transition by competitively binding to miR-124-3p in a hypoxic environment to regulate RELA expression and activate the downstream NF-κB pathway. Consequently, the PDIA3P1-miR-124-3p-RELA axis is a possible target for glioma therapy (88, 89). SChLAP1 forms a complex with HNRNPL to maintain the stability of ACTN4 and thus activates the NF-κB pathway to promote the growth of GBM cells (90). The identification of this complex provided a new perspective for the treatment of glioma. The UPF1-LINC00313-miR-342-3p/miR-485-5p-Zic4-SHCBP1 positive feedback loop was capable of modulating the biological behaviors of glioma cells, demonstrating that this loop is probably a potential therapeutic target (91). The combination of lncRNA and RBP forms a complex that regulates downstream target gene to contribute to TMZ resistance of glioma. This regulatory model provides a novel insight into the therapeutic strategy for glioma. Thus, targeting lncRNA-regulated angiogenesis, BTB permeability and TMZ resistance of glioma as novel strategies for the treatment of glioma shows potential.

## Conclusion

Over the previous decades, lncRNA studies have made major progress in the field of glioma research due to the rapid development of bioinformatics, and a series of lncRNAs have been found to act as indispensable factors in the occurrence and progression of glioma. LncRNAs act as ceRNAs in the cytoplasm to regulate glioma progression at the post-transcriptional level or to regulate gene expression through interactions with proteins in the nucleus. However, the mechanisms of most lncRNAs remain unclear. Therefore, the specific mechanisms of lncRNAs need to be further clarified. Glioma continues to be a major challenge to human health, and its advanced aggressiveness, chemoresistance and recurrence are the main factors contributing to the poor prognosis. Theoretically, there are numerous lncRNAs, such as HOTAIR, H19 and NEAT1, that can be applied as diagnostic and prognostic indicators of glioma. It is also possible that many lncRNAs can overcome TMZ resistance, modulate BTB permeability and control glioma angiogenesis, all of which are theoretically effective therapeutic strategies. Regrettably, no successful clinical use of lncRNAs has been achieved yet. In prospective research, lncRNA-centered gene regulatory networks should be constructed to elucidate the regulatory mechanisms of lncRNAs in tumor cells and then used as diagnostic, prognostic, and therapeutic indicators in clinical practice to improve the survival of glioma patients. One of the focuses of basic research is translation to the clinic to improve the survival of patients. Translating basic research into clinical strategies is a long road that will require generations of researchers to eventually understand the full picture of the function of lncRNAs in glioma at both the scientific and clinical levels.

## Author Contributions

JQ and XM wrote the draft and revised it. JC and CJ designed the tables. All authors contributed to the article and approved the submitted version.

## Funding

This study was supported by The National Natural Science Foundation of China (No. 81874204, No. 81772666, No. 81972817, No. 82073298, No. 82003022), Excellent Young Talents Project of Central Government Supporting Local University Reform and Development Fund (0202-300011190006), Karolinska Institutet Research Foundation Grants 2020-2021 (No. FS-2020:0007), The Heilongjiang Postdoctoral Science Foundation (LBH-Z18103, LBH-Z19029), and The Research Project of the Health and Family Planning Commission of Heilongjiang Province (2019-102).

## Conflict of Interest

The authors declare that the research was conducted in the absence of any commercial or financial relationships that could be construed as a potential conflict of interest.
